# von Economo neurones are selectively targeted in frontotemporal dementia

**DOI:** 10.1111/nan.12021

**Published:** 2013-07-09

**Authors:** A F Santillo, C Nilsson, E Englund

**Affiliations:** * Geriatric Psychiatric Unit, Department of Clinical Sciences, Lund UniversityLund, Sweden; † Department of Pathology, Lund University and Regional Laboratories Region SkåneLund, Sweden

**Keywords:** anterior cingulate cortex, frontotemporal dementia, frontotemporal lobar degeneration, von Economo neurones

## Abstract

**Background:**

von Economo neurones (VEN) are bipolar neurones located in the anterior cingulate cortex (ACC) and the frontoinsular cortex (FI), areas affected early in behavioural variant frontotemporal dementia (bvFTD), in which VEN may constitute a selectively vulnerable cellular population.

**Aim:**

A previous study has shown a selective loss of VEN in FTD above other neurones in the ACC of FTD. The aim of this study was to confirm this finding in a larger cohort, using a different methodology, and to examine VEN loss in relation to neuropathological severity and molecular pathology.

**Methods:**

VEN and neighbouring neurones (NN) were quantified in layers Va and Vb of the right dorsal ACC in 21 cases of bvFTD, 10 cases of Alzheimer's disease (AD) and 10 non-demented controls (NDC).

**Results:**

A marked VEN reduction was seen in all FTD cases. In the neuropathologically early cases of FTD (*n* = 13), VEN/10 000 NN was significantly reduced by 53% compared with NDC (*P* < 0.001) and 41% compared with AD (*P* = 0.019), whereas AD patients showed a non-significant 30% reduction of VEN/10 000 NN compared with NDC. VEN reduction was present in all protein pathology subgroups.

**Discussion:**

In conclusion, this study confirms selective sensitivity of VEN in FTD and suggests that VEN loss is an early event in the neurodegenerative process.

## Introduction

von Economo neurones (VEN) are large, bipolar projection neurones located almost exclusively in the anterior cingulate cortex (ACC) and the frontoinsula (FI) 1–3. Their function is unknown, but is hypothesized to include higher emotional cognitive integration 4. The distribution of VEN corresponds to that of the cortical regions first affected by atrophy in behavioural variant frontotemporal dementia (bvFTD) 5,6, a neurodegenerative disorder characterized by disruption of social, emotional and higher cognitive functioning 7. This led the group of Seeley 8 to examine if VEN were particularly affected in bvFTD. Using a stereological microscopic technique in seven cases of bvFTD, they showed a selective VEN reduction of 74% compared with neighbouring neurones in the ACC. This reduction was not seen in Alzheimer's disease (AD), in which VEN levels were comparable to that of controls 8. The same research group has shown selective VEN loss in the FI in nine cases of bvFTD compared with AD 9, although in this region it was not as severe and selective (55% reduction compared with controls, 43% reduction compared with AD). If these findings can be confirmed they will have important implications for the understanding of bvFTD pathobiology. If VEN are selectively targeted in bvFTD, it may explain why degeneration occurs particularly early in the ACC and FI before spreading into other cortical regions.

The molecular pathology underlying the neurodegeneration of bvFTD is heterogeneous, classified according to protein aggregation on neuropathological examination into primarily tau-positive cases, TDP-43 (TAR DNA binding protein 43)-positive cases and FUS (fused in sarcoma)-positive cases 10. VEN could constitute a common cellular end-point for these molecular processes. Also, if the symptoms in early bvFTD can be explained by early VEN density reduction, it may shed light on the functions of VEN, which are currently unknown. Several pieces of evidence point to a higher emotional–social function: especially their location in two areas of the paralimbic cortex which are commonly co-activated and implicated in higher cognitive emotional processes, such as self-awareness 11. The location of VEN overlaps with that of the salience network, which is thought to detect salient events and direct information to other cerebral networks, for higher order social emotional integration 12,13. VEN appear to have emerged late in phylogeny and are present only in humans, great apes and animals with more complex social interactions such as cetaceans and elephants 14–17, although their precise place in neuronal evolution is under examination 18. Their particular morphology suggests that they may be more vulnerable than other neurones, and not only to neurodegenerative disease processes. Consequently, pathophysiological roles for VEN have been suggested for several neurological and psychiatric diseases, and empirically studied in autism 19–21, schizophrenia 22 and corpus callosum agenesis 23. In autism, the findings have been heterogeneous, and possibly there are subgroups displaying both reduced and increased VEN density, whereas in schizophrenia, VEN density reduction seems to be a feature of early-onset cases.

The purpose of our study was to attempt to replicate the findings of selective VEN loss in the ACC in bvFTD 8 in a large number of clinically well-characterized cases, using a different methodological approach. If replicated, these findings would constitute an important advancement in our understanding of the pathophysiology of FTD, VEN and the FI/ACC system. In addition, we wished to study the relation between VEN loss, neuropathological severity and underlying molecular pathology.

## Material and methods

### Case selection

Selection criterion for FTD cases (*n* = 21; Table 1) was a neuropathological diagnosis of frontotemporal lobar degeneration (FTLD) 24,25 between the years 1994 and 2007 within the Department of Neuropathology in Lund and a clinical diagnosis of bvFTD. The presence of concomitant pathology was used as a neuropathological exclusion criterion. In particular, this was relevant for significant Alzheimer pathology, comprising neuronal loss, amyloid pathology and a neurofibrillary pathology at Braak stage >III 26, or significant cerebrovascular pathology, defined as vascular burden other than solitary microinfarcts. For the diagnosis of FTD clinical charts were reviewed and only patients fulfilling bvFTD as their first clinical syndrome, according to the 1998 criteria 27, were included. In these criteria the term behavioural variant FTD (bvFTD) is not used, only the older equivalent term FTD which corresponds to that of bvFTD, and these terms will be used interchangeably in this paper. Patients with a clinical onset of primary progressive aphasia were excluded, as were patients with evidence of a Parkinsonian syndrome (progressive supranuclear paralysis, corticobasal degeneration or other) during their clinical course. Individuals with concomitant motor neurone disease were not excluded. The FTD patients had been followed in a memory or neurology clinic, and the clinical investigations included structural neuroimaging, cerebral blood flow examination and neuropsychological examination in most cases. Stage of dementia was not assessed longitudinally and thus it was not possible to retrospectively determine the severity of dementia at death from the clinical records, but symptom duration in years was extracted. Patients with a neuropathological diagnosis of AD 26,28 (*n* = 10) were selected from the same time period, and matched to the FTD group by sex and age. AD cases with evidence of vascular pathology in the ACC were excluded. Non-demented controls (*n* = 10) were selected and matched to the neurodegenerative cases with regard to age and sex. They were required not to have a history of dementia or neuropathological signs of a dementia disorder. The neuropathological diagnosis was either tumour (4/10), cerebral ischaemia (4/10) or neurodegenerative disease without major cognitive deficits (multiple system atrophy, cerebellar ataxia), assessed <6 months prior to death (2/10). The study was approved by the Regional Ethical Review Board, Lund (Number 2010/229).

**Table 1:** Demographic data of cases and controls

	*n*	M/F	Age	Duration
FTD	21	11/10	62 (34–82)	7 (1–13)
Tau-pos	5	2/3	77 (49–80)	6 (3–12)
TDP-43-pos	14	8/6	69 (50–82)	6 (1–13)
FUS-pos	2	1/1	34, 37	3
AD	10	6/4	67 (57–76)	8 (4–14)
NDC	10	5/5	64 (54–82)	–

Age: median age in years, and range; duration: median duration from symptom onset until death, in years, and range.

M, male; F, female; FTD, behavioural variant frontotemporal dementia; tau-pos, tau-positive cases; TDP-43-pos, TAR DNA binding protein 43-positive cases and FUS-pos, fused in Sarcoma-positive cases, according to immunohistochemistry; AD, Alzheimer's disease; NDC, non-demented controls.

### Tissue preparation, staining and immunohistochemistry

From formalin-fixed, paraffin-embedded tissue blocks, whole brain coronal sections were taken, 6 μm thick, from a region immediately posterior to the anterior tip of the genu of the corpus callosum, thus yielding the ACC both ventrally and dorsally to the genu. The slices were stained with a double staining for nuclear material (Cresyl Violet/Nissl staining) and myelin (Luxol Fast Blue). Immunostaining methods that previously have been used to visualize VEN (neurofilament light protein NFL and microtubule-associated protein 2 MAP2) 3,4 were evaluated but in our hands none of these antibody stains allowed a thorough visualization of all neurones. The molecular pathology subtype of FTLD had been assessed prior to this study as part of the routine neuropathological examination and according to published criteria 25,29. We used these data as we did not aim to explore the spatial relationship between the pathological protein aggregates and VEN in this study. For FTLD molecular pathology subtype characterization, the following antibodies were used: AT-8 (for tau; DAKO, Copenhagen, Denmark), pTDP-43 (Cosmo Bio Ltd, Tokyo, Japan) and FUS (Sigma-Aldrich LLS, St Louis, MO, USA).

### Area delineation and neuropathological severity staging

**Figure 1:**
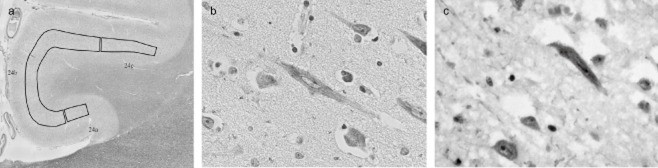
Region of interest deliniation and von Economo neurones morphology. (**a**) Coronal section of the right dorsal anterior cingulate cortex (Brodmann area 24, divided into subareas 24a, 24b and 24c) with cytoarchitectonical layers Va and Vb delineated, × 40. (**b**) Healthy von Economo neurone (VEN), × 200. (**c**) Dystrophic VEN in a case of frontotemporal dementia, × 200. All stained with Cresyl Violet/Nissl staining and myelin (Luxol Fast Blue) on paraffined sections, 6 μm thick.

The ACC was defined as the cortical fold adjacent to the genu, regardless of cingulate/paracingulate cortex variability. The dorsal ACC of the right hemisphere was chosen as VEN density shows lateralization, with slight right to left hemispheric preponderance 14. The entire right dorsal ACC was scanned on a motorized light microscope at × 200 magnification (Olympus BX53; Olympus Europe Group, Hamburg, Germany), using one section per individual (Figure 1a). On digitalized scans the layer V (Va and Vb) was manually outlined and further subdivided into the cytoarchitectural subregions 24a, 24b and 24c according to established criteria 30 (Figure 1a). In this study we examined only subarea 24b, because this possibly lowers variability of VEN density. 24b is the largest subregion and has the highest VEN density 1, while 24c has the lowest density. As the VEN density over the whole gyrus will be dependent on the relative amounts of 24b and 24c (and 24a, but this area is considerably smaller), using only 24b instead of the whole gyrus was estimated to lower the variability. Area 24b in our cases always included both ‘crowns’ of the gyrus, which have particularly high VEN densities 2,14 (Figure 1a). Neuropathological severity of disease was assessed using the rating scale developed by Broe *et al*. 6. Briefly this validated scale divides FTD cases in stages 1 to 4 depending on macroscopic atrophy of frontal cortical, limbic and subcortical areas, examined on two coronal whole-brain slides. As an additional measure of neuropathological severity, the maximum cortical thickness of the dorsal ACC (in μm) was measured in each case.

### Cell counting

Cells were counted on the digitalized scans of area 24b of the dorsal ACC, with systematic scanning performed twice through the area at × 200 magnification. VEN were identified on the basis of established morphological criteria 15: a long, slender, elongated soma, with one apical and one basal process of approximately the same size extending along the main axis of the soma. The cell and its processes had to be aligned along the main cellular direction of the surrounding tissue. VEN were required to have at least the same size as layer V pyramidal neurones. Visibility of the nucleus and/or the nucleolus was not a criterion, which is an approach we chose to avoid floor effects in cases with severe VEN loss. Care was taken not to count layer VI spindle neurones that sometimes are found in layer Vb. VEN on the border of the delineated areas were counted if more than 50% of their length were inside the area. In the same area of each case (Va and Vb of 24b), normal neurones (NN, excluding VEN) were also counted. NN were counted in order to be able to provide a ratio between VEN and NN, our main outcome. This approach has two possible advantages, firstly, to avoid a problem in quantifying neurones in neurodegenerative disease, which is disproportionate reduction in area which distorts cellular density (number/area) measurements 8. Secondly, normalizing VEN/NN allows for comparisons of the relative VEN loss between FTD and AD, as the two conditions have a different propensity for layer V neurones. Criteria for NN were a visible and intact nucleus and nucleolus, a clear soma (but intactness was not a requirement), and not having typical glial appearance. NN on the border of the delineated area were not counted. All cell counting (VEN and NN) was done blinded for diagnosis and all other information on the case.

After thorough training and experimentation with different sets of criteria, interrater and intrarater reliability for the cell counting procedure described was assessed by counting VEN and NN in 10 cases (five patients, five controls) by two of the authors (A. S. and E. E., A. S. *vs.* A. S. and A. S. *vs.* E. E.). Reliability was calculated using the intraclass correlation coefficient (ICC) (2.1, absolute agreement, single measures, two-way mixed model). Interrater reliability for VEN counting was 0.977 (0.664–0.996, *P* < 0.001) and intrarater 0.967 (0.865–0.992, *P* < 0.001) respectively. For NN the intrarater reliability was 0.982 (0.914–0.996, *P* < 0.001) and the interrater 0.896 (0.727–0.989, *P* < 0.001).

### Statistical analysis

To ascertain possible differences in age and sex distribution in FTD, AD and NDC groups were compared with the Kruskal–Wallis test (independent sample) for age, and the exact χ^2^-test (with significance set to 0.05) for sex distribution. Number of VEN was normalized to VEN/10 000 NN of each 24b. The primary outcome was mean VEN/10 000 NN in subregion 24b compared between FTD, AD and HC. For this comparison, one-way analysis of variance (anova) with Tukey's *post hoc* test was used, with the statistical significance set to 0.05 in each comparison. Q–Q plot analysis and the Kolmogorov–Smirnov test (*P* = 0.200) showed that parametric analysis could be used for our data. VEN/10 000 NN in the FTD group were divided according to neuropathological stage and molecular neuropathological status and compared using a two-tailed Student's *t*-test, with significance set to 0.05. For all correlations a two-tailed Pearson's test of correlation was employed, also with *P* set to 0.05.

## Results

Twenty-one cases of FTD (median age 62, 11 men, 10 women), 10 cases of AD (median age 67.5, six men, four women) and eight NDC (median age 62, four men, four women) were included in the study (Table 1). There were no significant differences in age (*P* = 0.861) or sex distribution (χ^2^ = 1.425, *P* = 0.295). Of the 21 FTD cases, five were tau-positive and 16 tau-negative, and of the latter 14 were TDP-43-positive and two FUS-positive (Table 1). Five of the FTD cases were of neuropathological severity stage 1, eight cases at stage 2, four at stage 3 and four at stage 4.

**Figure 2:**
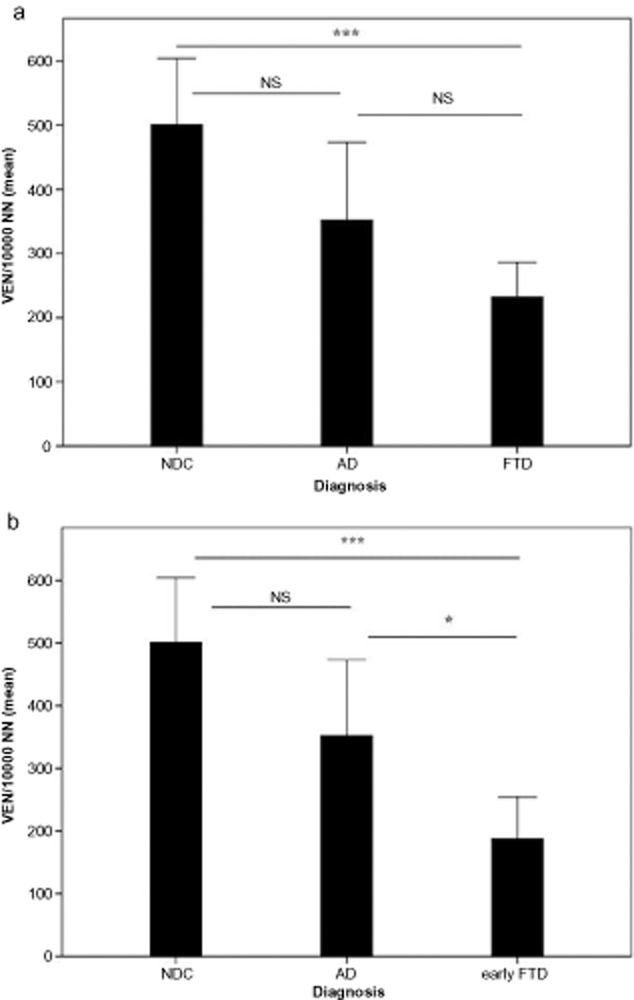
von Economo neurones as a function of diagnosis. (**a**) Mean von Economo neurones (VEN)/10 000 neighbouring neurones (NN) in non-demented controls (NDC), patients with Alzheimer's disease (AD) and frontotemporal dementia (FTD). (**b**) Cases of neuropathologically early FTD (Broe stages 1 and 2) compared with NDC and patients with AD. *P < 0.05 level; ***P < 0.001; NS, not significant. Bars represent 95% confidence interval.

**Figure 3:**
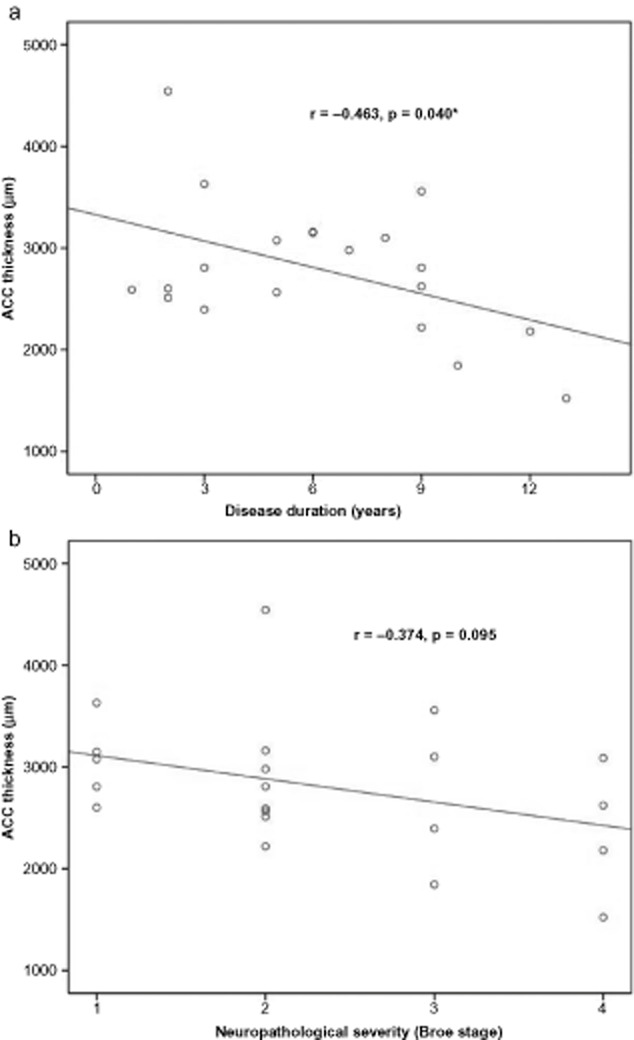
Relationship between morphology, disease duration and severity in cases of frontotemporal dementia. (**a**) Correlation between anterior cingulate cortex (ACC) thickness (in μm) and disease duration (in years) (*r* = −0.463, *P* = 0.040). (**b**) Correlation between neuropathological severity (stages 1–4 according to Broe) and ACC thickness (in μm) (*r* = −0.374, *P* = 0.095). *Statistical significance at the 0.05 level. Correlation coefficient according to Pearson.

On average NDC patients had 501 VEN/10 000 NN [95% confidence interval (CI) 398–604], while FTD patients showed 54% less VEN/10 000 NN with a mean of 233 (95% CI 179–286), whereas AD patients had mean VEN/10 000 NN of 352 (95% CI 231–473) (Figure 2a), a 30% reduction. The effect of diagnosis was statistically significant (*F* = 12.1, *P* < 0.001), with the difference between FTD and NDC being statistically significant (*P* < 0.001), while the difference between AD and NDC (*P* = 0.062) or AD and FTD (*P* = 0.064) was not (Figure 2a). VEN density in the FTD group differed according to neuropathological severity stage with stages 1 and 2 showing lower VEN levels (*n* = 13, 198 and 187 VEN/10 000 NN) than 3 and 4 (*n* = 8, 321 and 290 VEN/10 000 NN) (Student's *t*-test *P* = 0.020). A repeated anova with VEN/10 000 NN of stage 1 and 2 (mean VEN/10 000 NN 188, 95% CI 121–254) showed a significant effect of diagnosis (*F* = 13.8, *P* < 0.001) with VEN/10 000 NN in FTD being significantly lower than both NDC (53% reduction, *P* < 0.001) and AD (41%, *P* = 0.019) (Figure 2b). In accordance with this finding of lower VEN/10 000 NN in neuropathologically earlier stages a moderate and inverse relationship between macroscopic severity and VEN density was found when ACC thickness was correlated with VEN/10 000 NN, with *r*_s_ = −0.42 and borderline significance (*P* = 0.058). As expected, disease duration and ACC thickness exhibited an inverse significant correlation (*r* = −0.463, *P* = 0.040) (Figure 3a), with the more neuropathologically severe stage having thinner ACC (*r* = 0.374) (Figure 3b), although this was not statistically significant (*P* = 0.095). The VEN in FTD revealed a classical appearance, but also a dystrophic (pyknotic) state, as previously described (Figure 1c) 8. Table 2 shows the absolute number of VEN/24b in each section, which are significantly reduced in FTD compared with NDC (−48%, *P* = 0.007) but not in AD (−32%, *P* = 0.118) compared with NDC. The difference between FTD and AD (24% less VEN in FTD) was not statistically significant (*P* = 0.518). Dividing VEN density by molecular neuropathology showed lower VEN/10 000 NN across all molecular subtypes with tau-positive cases (*n* = 5) having 208 VEN/10 000 NN and tau-negative (*n* = 16) 240 VEN/10 000 NN. The latter are divided into TDP-43-positive cases (*n* = 14) with 239 VEN/10 000 NN and FUS-positive cases (*n* = 2) with 180 VEN/10 000 NN. Both the tau- and FUS-positive groups were too small for statistical group comparisons; however, there were no statistically significant differences between the three different molecular subtypes (*F* = 0.406, *P* = 0.672 with anova). The TDP-43-positive group showed a significant reduction of VEN/10 000 NN when compared with NDC (*P* = 0.001) but not with AD (*P* = 0.083).

**Table 2:** VEN/10 000 NN: mean VEN normalized to 10 000 NN, in the same area

	VEN/10 000 NN	VEN/24b
NDC	501 (398–604)	36.6 (23.9–49.3)
AD	352 (231–473)	24.9 (11.7–38.0)
FTD	233 (179–286)	18.9 (13.7–23.9)*

95% confidence intervals within brackets.

* Mean VEN/section in FTD is statistically different compared with NDC (*P* = 0.007) but not with AD (*P* = 0.118).

VEN, von Economo neurones; NN, normal neurones; VEN/24b, mean absolute number of VEN per section, counted in layers Va and b of area 24b; NDC, non-demented controls; AD, Alzheimer's disease; FTD, frontotemporal dementia.

## Discussion

In this study we show that the neurodegenerative process of FTD is associated with a 53% greater loss of VEN than of neighbouring layer V neurones, suggesting that VEN are more sensitive to the neurodegenerative process in FTD than other types of neurones, or the neurodegenerative process in Alzheimer's disease. This is in accordance with previous findings and is the first study to replicate the findings of Seeley *et al*. of selective VEN loss in early bvFTD 8. Our study included a considerably larger number of cases, and applied a different methodology. In our study, the relative VEN loss in the ACC was somewhat less pronounced than that seen in the previous study (74%), having more resemblance to the results reported for the FI (56%) 9. In AD, we could detect a 32% (not statistically significant) VEN reduction, which is in line with the results of the Seeley group in the FI, whereas in their study on the ACC, VEN levels in AD were close to that of controls. These differences may be due to different methodologies but could also be due to case selection, in view of the relatively small number of cases included. Another explanation is that while VEN loss seems to be a particular feature in FTD, it may not be as exclusive for this neurodegenerative process as previously thought.

Our findings show that earlier FTD stages had lower VEN/10 000 NN than more severe stages, a pattern also seen when VEN density was correlated with ACC atrophy. The finding of a relatively more marked VEN loss in neuropathologically early cases *vs.* late cases is in line with the two previous studies examining VEN in FTD, where Broe stage 3 has a slightly higher VEN/NN density than stage 2 8,9. One could expect the discrepancy between VEN and NN loss to increase as the disease progresses. We hypothesized that possible sources of bias in our methodology could exaggerate relative VEN loss in more advanced cases. Due to the collapse of cortical layers in advanced cases VEN may lose perpendicularity and hence may not be counted. The risk of counting glial cells as NN in those cases may have lowered the VEN numbers. On the other hand, in severe cases distinction between dystrophic VEN and layer VI spindle neurones may be more difficult. If this finding is true, it appears that VEN are selectively vulnerable to the early disease processes in FTD, but as the disease progresses other neurones in layer V will be increasingly affected, explaining the higher VEN/10 000 NN ratio in these cases. This corresponds to the notion that FTD pathology initially affects superficial cortical layers while deeper layers are affected as the disease progresses 31. It is also possible that patients with a lower degree of macroscopic atrophy at the time of death represent a population of FTD patients different from FTD patients with greater degrees of atrophy at the time of death. As the clinical disease severity at the time of death has not been determined in this study this hypothesis cannot be confirmed at present, but one might speculate that early loss of VEN leads to more rapid functional decline and an earlier death with less macroscopic atrophy.

Methodologically, given that the whole cingulate gyrus was visible for the rater, there may have been a suboptimal blinding, probably not so for AD *vs.* FTD but perhaps for NDC *vs.* severe cases of AD/FTD, even though our NDC were not ‘super controls’ and displayed different signs of pathology. This possible loss of blinding should be less of a problem with early cases, which in our sample demonstrated the greatest relative loss of VEN. Light microscopy, as employed in our study, has the advantage of being less time-consuming, but may be less accurate compared with stereological methods in terms of both reliability and validity. For VEN quantification, light microscopy has been used before in the study of schizophrenia 22. In our NDC group, the VEN represent about 5% of the total neurone number in layer V which is in the same magnitude as in studies using stereology: 1.5% in Seeley *et al*. 8, and 2–3% in Kim *et al*. 9. We think that the higher percentage VEN/NN in our study mirrors the fact that we only studied area 24b, which has the highest VEN density, and because we consciously applied slightly more generous VEN criteria *vs.* NN criteria than previous studies (a visible and intact nucleus and/or the nucleolus was not a criterion in our study). VEN are not uniformly distributed in the cortex but instead vary in density along the gyrus, even in the same subarea (higher density in the ‘crowns’, lower in the flats of 24b, for example), and often show clustering in the layer where they are found, layer V. In such a situation with high variability, covering large areas as with light microscopy could enable a more comprehensive estimation 32. The variability in our measurement is not higher in our study than in studies using stereology 8,9 (one standard error of the mean is 8.8% of the mean value in our study, compared with approximately 33% and 12% respectively). In the context of our objective, which was to replicate a previous finding, the application of a different method is an advantage.

Clearly, there can be a discrepancy between neuropathologically early FTD and clinically early FTD. We do not have a clinical longitudinal disease severity assessment of our cohort but our neuropathologically early cases (stages 1 and 2) had a disease duration from symptom onset of 4.6 years and do include several cases with a disease duration from symptom onset of 2 years with definite lower VEN/NN levels. As in previous studies we show that selective VEN loss seems to be a feature of all molecular pathology subtypes, although the numbers of tau- and FUS-positive cases in our study are small. This indicates that VEN could constitute a common cellular end-point of the diverse molecular pathologies and genetic aberrations underlying FTD. The neurodegenerative process could then subsequently spread to neighbouring neurones, and thus explain the propensity of bvFTD for ACC and FI. Alternatively, the pathogenetic process may have selectivity for ACC and FI that is not directly related to VEN, but these cells are more susceptible to this process and contribute to the symptoms of FTD.

In conclusion, our study replicates the previous findings of selective VEN loss in early bvFTD. The contribution of VEN loss relative to loss of other neurones is yet to be explored. There is a parallel loss of layer V neurones, which certainly also has pathophysiological significance. We have made no comparison between VEN and neurones of superficial layers II and III, which generally are more affected in FTD than layers V and VI 31, a comparison which would be suitable for further studies as well as that of the contribution of VEN loss to specific symptoms of FTD.

## References

[1] Nimchinsky EA, Vogt BA, Morrison JH, Hof PR (1995). Spindle neurons of the human anterior cingulate cortex. J Comp Neurol.

[2] von Economo K (1925). Die Cytoarchitektonik der Hirnrinde des Erwachsenen Menschen.

[3] Fajardo C, Escobar MI, Buritica E, Arteaga G, Umbarila J, Casanova MF, Pimienta H (2008). Von Economo neurons are present in the dorsolateral (dysgranular) prefrontal cortex of humans. Neurosci Lett.

[4] Allman JM, Watson KK, Tetreault NA, Hakeem AY (2005). Intuition and autism: a possible role for Von Economo neurons. Trends Cogn Sci.

[5] Seeley WW, Crawford R, Rascovsky K, Kramer JH, Weiner M, Miller BL, Gorno-Tempini ML (2008). Frontal paralimbic network atrophy in very mild behavioral variant frontotemporal dementia. Arch Neurol.

[6] Broe M, Hodges JR, Schofield E, Shepherd CE, Kril JJ, Halliday GM (2003). Staging disease severity in pathologically confirmed cases of frontotemporal dementia. Neurology.

[7] Piguet O, Hornberger M, Mioshi E, Hodges JR (2011). Behavioural-variant frontotemporal dementia: diagnosis, clinical staging, and management. Lancet Neurol.

[8] Seeley WW, Carlin DA, Allman JM, Macedo MN, Bush C, Miller BL, Dearmond SJ (2006). Early frontotemporal dementia targets neurons unique to apes and humans. Ann Neurol.

[9] Kim EJ, Sidhu M, Gaus SE, Huang EJ, Hof PR, Miller BL, DeArmond SJ, Seeley WW (2012). Selective frontoinsular von Economo neuron and fork cell loss in early behavioral variant frontotemporal dementia. Cereb Cortex.

[10] Rademakers R, Neumann M, Mackenzie IR (2012). Advances in understanding the molecular basis of frontotemporal dementia. Nat Rev Neurol.

[11] Craig AD (2009). How do you feel—now? The anterior insula and human awareness. Nat Rev Neurosci.

[12] Sridharan D, Levitin DJ, Menon V (2008). A critical role for the right fronto-insular cortex in switching between central-executive and default-mode networks. Proc Natl Acad Sci U S A.

[13] Cauda F, Torta DM, Sacco K, D'Agata F, Geda E, Duca S, Geminiani G, Vercelli A (2013). Functional anatomy of cortical areas characterized by Von Economo neurons. Brain Struct Funct.

[14] Allman JM, Tetreault NA, Hakeem AY, Manaye KF, Semendeferi K, Erwin JM, Park S, Goubert V, Hof PR (2010). The von Economo neurons in frontoinsular and anterior cingulate cortex in great apes and humans. Brain Struct Funct.

[15] Nimchinsky EA, Gilissen E, Allman JM, Perl DP, Erwin JM, Hof PR (1999). A neuronal morphologic type unique to humans and great apes. Proc Natl Acad Sci U S A.

[16] Butti C, Sherwood CC, Hakeem AY, Allman JM, Hof PR (2009). Total number and volume of Von Economo neurons in the cerebral cortex of cetaceans. J Comp Neurol.

[17] Hakeem AY, Sherwood CC, Bonar CJ, Butti C, Hof PR, Allman JM (2009). Von Economo neurons in the elephant brain. Anat Rec (Hoboken).

[18] Evrard HC, Forro T, Logothetis NK (2012). Von Economo neurons in the anterior insula of the macaque monkey. Neuron.

[19] Simms ML, Kemper TL, Timbie CM, Bauman ML, Blatt GJ (2009). The anterior cingulate cortex in autism: heterogeneity of qualitative and quantitative cytoarchitectonic features suggests possible subgroups. Acta Neuropathol (Berl).

[20] Santos M, Uppal N, Butti C, Wicinski B, Schmeidler J, Giannakopoulos P, Heinsen H, Schmitz C, Hof PR (2011). Von Economo neurons in autism: a stereologic study of the frontoinsular cortex in children. Brain Res.

[21] Kennedy DP, Semendeferi K, Courchesne E (2007). No reduction of spindle neuron number in frontoinsular cortex in autism. Brain Cogn.

[22] Brune M, Schobel A, Karau R, Benali A, Faustmann PM, Juckel G, Petrasch-Parwez E (2010). Von Economo neuron density in the anterior cingulate cortex is reduced in early onset schizophrenia. Acta Neuropathol (Berl).

[23] Kaufman JA, Paul LK, Manaye KF, Granstedt AE, Hof PR, Hakeem AY, Allman JM (2008). Selective reduction of Von Economo neuron number in agenesis of the corpus callosum. Acta Neuropathol (Berl).

[24] Cairns NJ, Bigio EH, Mackenzie IR, Neumann M, Lee VM, Hatanpaa KJ, White CL, Schneider JA, Grinberg LT, Halliday G, Duyckaerts C, Lowe JS, Holm IE, Tolnay M, Okamoto K, Yokoo H, Murayama S, Woulfe J, Munoz DG, Dickson DW, Ince PG, Trojanowski JQ, Mann DM (2007). Neuropathologic diagnostic and nosologic criteria for frontotemporal lobar degeneration: consensus of the Consortium for Frontotemporal Lobar Degeneration. Acta Neuropathol (Berl).

[25] Mackenzie IR, Neumann M, Bigio EH, Cairns NJ, Alafuzoff I, Kril J, Kovacs GG, Ghetti B, Halliday G, Holm IE, Ince PG, Kamphorst W, Revesz T, Rozemuller AJ, Kumar-Singh S, Akiyama H, Baborie A, Spina S, Dickson DW, Trojanowski JQ, Mann DM (2010). Nomenclature and nosology for neuropathologic subtypes of frontotemporal lobar degeneration: an update. Acta Neuropathol (Berl).

[26] Braak H, Alafuzoff I, Arzberger T, Kretzschmar H, Del Tredici K (2006). Staging of Alzheimer disease-associated neurofibrillary pathology using paraffin sections and immunocytochemistry. Acta Neuropathol (Berl).

[27] Neary D, Snowden JS, Gustafson L, Passant U, Stuss D, Black S, Freedman M, Kertesz A, Robert PH, Albert M, Boone K, Miller BL, Cummings J, Benson DF (1998). Frontotemporal lobar degeneration: a consensus on clinical diagnostic criteria. Neurology.

[28] Brunnstrom H, Englund E (2011). Comparison of four neuropathological scales for Alzheimer's disease. Clin Neuropathol.

[29] Mackenzie IR, Neumann M, Baborie A, Sampathu DM, Du Plessis D, Jaros E, Perry RH, Trojanowski JQ, Mann DM, Lee VM (2011). A harmonized classification system for FTLD-TDP pathology. Acta Neuropathol (Berl).

[30] Vogt BA, Nimchinsky EA, Vogt LJ, Hof PR (1995). Human cingulate cortex: surface features, flat maps, and cytoarchitecture. J Comp Neurol.

[31] Brun A (1987). Frontal lobe degeneration of non-Alzheimer type. I. Neuropathology. Arch Gerontol Geriatr.

[32] Benes FM, Lange N (2001). Two-dimensional versus three-dimensional cell counting: a practical perspective. Trends Neurosci.

